# Predator Exposure/Psychosocial Stress Animal Model of Post-Traumatic Stress Disorder Modulates Neurotransmitters in the Rat Hippocampus and Prefrontal Cortex

**DOI:** 10.1371/journal.pone.0089104

**Published:** 2014-02-14

**Authors:** C. Brad Wilson, Philip J. Ebenezer, Leslie D. McLaughlin, Joseph Francis

**Affiliations:** 1 Comparative Biomedical Sciences, Louisiana State University, School of Veterinary Medicine, Baton Rouge, Louisiana, United States of America; 2 Pathobiological Sciences, Louisiana State University, School of Veterinary Medicine, Baton Rouge, Louisiana, United States of America; Universidade de São Paulo, Brazil

## Abstract

Post-Traumatic Stress Disorder (PTSD) can develop in response to a traumatic event involving a threat to life. To date, no diagnostic biomarkers have been identified for PTSD. Recent research points toward physiological abnormalities in the hypothalamic-pituitary-adrenal (HPA) axis, sympathoadrenal medullary and immune system that may be implicated in the disorder. The modulation of neurotransmitters is another possible mechanism, but their role in the progression of PTSD is poorly understood. Low serotonin (5-HT) may be a factor, but it may not be the only neurotransmitter affected as modulation affects levels of other neurotransmitters. In this study, we hypothesized the predator exposure/psychosocial stress rodent model of PTSD may alter levels of 5-HT and other neurotransmitters in the rat hippocampus and prefrontal cortex (PFC). Male Sprague-Dawley rats were used in this experiment. We induced PTSD via a predator exposure/psychosocial stress model, whereby rats were placed in a cage with a cat for 1 hour on days 1 and 11 of the 31-day experiment. Rats also received psychosocial stress via daily cage cohort changes. On day 32, the rats were sacrificed and the brains dissected to remove the hippocampus and PFC. Norepinephrine (NE), 5-Hydroxyindoleacetic acid (5-HIAA), homovanillic acid (HVA), dopamine (DA), and 3,4-Dihydroxyphenylacetic acid (DOPAC), and 5-HT levels in the hippocampus and PFC were measured with high-performance liquid chromatography (HPLC). In the hippocampus, 5-HT and HVA were lower, while NE and DOPAC were higher, in the PTSD group vs. controls. In the PFC, only 5-HT was lower, while NE, DA, and DOPAC were higher, in the PTSD group vs. controls. The rate limiting enzymes tyrosine hydroxylase and tryptophan hydroxylase were also examined and confirmed our findings. These results demonstrate that the predator exposure/psychosocial stress model of PTSD produces neurotransmitter changes similar to those seen in human patients and may cause a heightened noradrenergic response.

## Introduction

Post-Traumatic Stress Disorder (PTSD), recently reclassified as a trauma- and stressor-related disorder, can develop in response to real or perceived life-threatening situations. According to the Diagnostic and Statistical Manual of Mental Disorders 5 (DSM-5), a diagnosis of PTSD necessitates exposure to a life-threatening event, intrusive recollections of the event, avoidance of associated stimuli and numbing of general responsiveness, negative cognitions/mood, hyperarousal not present before the trauma, and a significant social impairment. All of these symptoms must persist for at least 30 days and not be due to illness, medication, or substance abuse [1]. To date, no definitive diagnostic biomarkers have been identified for PTSD. Recent research, however, points toward physiological abnormalities in the hypothalamic-pituitary-adrenal (HPA) axis, sympathoadrenal medullary system, and immune system that may be implicated in the disorder[2]–[5]. In the brain, neurotransmitter modulation may also play a critical role in PTSD development, and they continue to be the primary target for pharmacologic interventions. It still remains unclear, however, exactly which neurotransmitters are up- or down-regulated during PTSD progression.

A growing body of evidence suggests that exposure to traumatic stressors and psychological trauma may result in increased morbidity and mortality. Much of the data available suggest traumatic exposure and subsequent PTSD may lead to increased incidence of cardiovascular disease, diabetes, chronic fatigue syndrome, and other conditions[6]–[9], but the involvement of neurotransmitters has yet to be clearly delineated. Serotonin (5-HT), for example, is a neurotransmitter responsible for many functions in the central nervous system (CNS) and peripheral organs. 5-HT influences aggression, arousal, sleep, anxiety, appetite, fear, learning, and other actions [10]. 5-HT is also the principle regulator of mood. A study by Peirson et al. [11] found lower platelet 5-HT_2_ receptor function was associated with depressed mood, while Williams et al. [12] demonstrated higher blood 5-HT levels were correlated with better mood. An increased mood and overall sense of well-being has been shown, in both psychiatric and physical disorders, as protective and positively correlated with resiliency behavior [13]. PTSD research has demonstrated that 5-HT-uptake sites in platelets were lower in PTSD patients vs. controls [14]. Lower 5-HT has also been implicated in diminished physical health. Muldoon et al. showed that a low prolactin response to fenfluramine, a drug that increases 5-HT levels, was associated with metabolic syndrome [15].

Norepinephrine (NE), a neurotransmitter principally affecting excitatory receptors, is also involved in the regulation of psychiatric and physical mechanisms. Under normal conditions, NE is a principle component of the stress response, directly increasing heart rate and blood flow to skeletal muscles and triggering the release of glucose, all in preparation for the ‘fight-or-flight’ response. Persistent noradrenergic activity, however, has been linked with negative outcomes in patients with congestive heart failure (CHF) [16] and diabetes [17]. Studies have also shown that individuals with PTSD have elevated cerebrospinal fluid (CSF) levels of NE [18] and noradrenergic hyperresponsiveness to various stimuli [3]. Dysregulation of noradrenergic neurons has also been associated with hyperarousal and intrusive recollections attributable to PTSD [19].

Dopamine (DA), a neurotransmitter with primarily inhibitory effects, plays a major role in emotion and the reward system of the brain. It optimally functions within a narrow range and dopaminergic hypo- or hyperactivity is implicated in both physical and psychiatric illnesses. Parkinson’s disease is characterized by a loss of dopaminergic neurons, and evidence suggests schizophrenia and psychosis are linked to elevated levels of DA [20]. DA may also have a role in PTSD, and studies have shown dopaminergic hyperactivity in male combat veterans [21], traumatized adult females [22], and abused children [23] with PTSD. The dopamine metabolite homovanillic acid (HVA), often used as a diagnostic test for catecholamine-producing tumors of the adrenal glands, has also demonstrated aberrant levels in PTSD patients. Geracioti et al. found HVA was significantly reduced in the CSF of combat-related PTSD patients immediately after viewing traumatic imagery [24]. Based on the previous research, the primary focus of this study was to determine how neurotransmitters were modulated in response to a predator exposure/psychosocial stress rodent model of pre-clinical PTSD.

## Materials and Methods

### Ethics Statement

This study was carried out in strict accordance with the recommendations of the Institute for Laboratory Animal Research’s 2011 *Guide for the Care and Use of Laboratory Animals*, under the auspices of an animal care and use protocol approved by the Louisiana State University Institutional Animal Care and Use Committee (Protocol Number: 12-067). Upon completion of all PTSD-related experiments and in adherence with the approved protocol, the cats will be adopted out to approved families in the local area.

### Animals

Naïve adult male Sprague-Dawley rats (Harlan Laboratories, Indianapolis, IN) were used in all experiments. The rats were the same age (12 weeks) and approximately the same weight (±15 g) upon delivery. Rats were pair-housed in standard plastic microisolator cages and had access to food and water *ad libitum*. The cages were maintained in ventilated racks (racks hold eight cages vertically and five horizontally) and each cage was randomly assigned to a specific rack location to ensure groups were evenly distributed. The vivarium room was kept on a 12-hour light/dark cycle (0700–1900), room temperature was maintained at 20±1°C, and humidity ranged from 23–42%. After a one-week acclimation period, the mean weight of all rats was 347.9 g ±4.5. Two cats, one male and one female (Harlan Laboratories, Indianapolis, IN (male), and Tulane University, New Orleans, LA (female)) were used for all predator exposures. Cats were seven and ten years old, respectively. They were housed in an open room (15′×15′) in the vivarium with access to food, water, and enrichment devices *ad libitum*. The cat room was on the same light/dark cycle and maintained at similar temperature and humidity as the rat room.

### Stress Induction

Following the acclimation period, rats were brought to the laboratory and under isoflurane anesthesia were weighed, ear-tagged, tail-marked (ear tag number written on tail for easy identification), and 250–500 µL of blood was drawn from either the tail or lateral saphenous vein. The rats were then randomly assigned to the PTSD (n = 10) or control (n = 10) group and returned to the vivarium for 24 hours. The following day, PTSD rats were started on a predator exposure/psychosocial stress regimen, published and validated by Zoladz et al., designed to produce a pre-clinical PTSD that closely mimics signs and symptoms seen in human patients [25], [26]. Briefly, PTSD rats were individually isolated in cylindrical, Plexiglas containers (IITC Life Science, Inc., Woodland Hills, CA; tail cuff restraint containers for 400–600 g rats and Kent Scientific, Torrington, CT; tail cuff restraint containers for 300–500 g rats) and canned cat food (Friskies, Purina, St. Louis, MO) was smeared on the outside of the cylinders. The cylinders prevented direct contact with the cats, and the cat food induced predatory movement in the cats. Studies show a moving cat invokes a greater fear response than a sedentary cat [27]. Rats were then placed in a stainless steel holding cage (76 cm×76 cm×60 cm) consisting of a solid metal floor with a hinged, metal rod door, with a cat for one hour. The first cat exposure was conducted during the light cycle (0700–1900). Ten days later, a second cat exposure was conducted during the dark cycle (1900–0700). In addition to the cat exposures, starting on day one the rats were subjected to psychosocial stress by changing their cage cohort daily. The cage cohort rotation was established prior to the start of the experiment, whereby each rat was never housed with the same rat on consecutive days and never housed with the same rat more than four times in a month. The predator exposure/psychosocial stress regimen was continued for 31 days. After 31 days, PTSD and control group rats were euthanized via decapitation and the brains were immediately removed. The hippocampus, amygdala, and prefrontal cortex (PFC) were dissected and flash-frozen in liquid nitrogen.

### High-Performance Liquid Chromatography (HPLC)

#### HPLC – preparation of standard solution

Neurotransmitter concentrations were detected using Eicom HTEC 500 high performance liquid chromatography system. The standard solutions of norepinephrine (NE; MW 337.3), 3, 4- dihydroxyphenylacetic acid (DOPAC; MW 168.15), dopamine (DA; MW 158.17), 5-Hydroxyindoleacetic acid (5-HIAA; MW 218.68), homovanillic acid (HVA; MW 182.18), 5-Hydroxytryptamine (5-HT; MW 212.68) and Isoproterenol (internal standard; MW 247.7), each 1 ng/µL concentration, were prepared by serial dilution. 5-HT and 5-HIAA were dissolved in 0.1 M acetic acid including 1 mg/mL EDTA and other salts were prepared in 0.1 M hydrochloric acid including 1 mg/mL EDTA. These solutions were prepared and filtered through a 0.45 µm centrifuge tube filter before injection into the HPLC system. Different concentrations were injected by maintaining the volume of injection at 10 µL in order to quantify sample values after authenticating the retention time of individual neurotransmitters.

#### HPLC – preparation of samples

Sample preparations from the experimental animals were carried out according to the procedure of Deyama et al. [28]. Hippocampus and PFC tissue were weighed and dissected before homogenizing at 4°C with 0.2 M perchloric acid including 100 uM EDTA-2Na in a Teflon/glass homogenizer. The homogenate was centrifuged at 4°C for 15 min at 20,000×g. The supernatant was collected and filtered through a 0.45 µm centrifuge tube filter before injection into the HPLC system.

#### HPLC – detection of neurotransmitters

The following working conditions were maintained in the HPLC system: isocratic elution; mobile phase (Citrate buffer in methanol with EDTA and sodium Octane sulfonate); Eicompak SC-3ODS (ID 3.0×100 mm) column; flow rate 340 µL/min; graphite working electrode WE-3G (Gasket GS-25), (+750 mV versus Ag/AgCl electrode); temperature 25°C. The levels of neurotransmitters are expressed as pg/µg of wet tissue.

#### HPLC – mobile phase

Citric acid monohydrate (8.84 g; mol wt. 210.14), and 3.10 g of sodium acetate (mol. wt. 82.03) in 800 ml of MilliQ Ultrapure fresh water (>18.2 MΩ/cm) and 200 ml of HPLC grade methanol were added and shaken well (magnetic stirrer not used). EDTA (Dojindo Laboratories, USA, mol. wt. 372.24; 0.005 g) and sodium octane sulfonate (Dojindo Laboratories, USA, and 0.220 g) were added and shaken well.

### Western Blot

Tissue homogenates from the hippocampus and PFC were subjected to Western Blot (WB) analysis (n = 10/group) for the determination of protein levels of the norepinephrine and dopamine rate-limiting enzyme tyrosine hydroxylase (TH), the 5-HT rate-limiting enzyme tryptophan hydroxylase (TPH), and GAPDH. The extraction of protein and WB was performed as previously described [29]. The specific antibodies used included: TH, TPH, and GAPDH. Primary antibodies were commercially obtained: TH, 1∶1000 dilution (AB-112, Abcam, Cambridge, MA.); TPH, 1∶1000 dilution (AB-1541, Millipore, Billerica, MA.); GAPDH, 1∶1000 dilution (SC-25778, Santa Cruz Biotechnology, Santa Cruz, CA). Secondary antibodies were commercially obtained: anti-rabbit, 1∶5000 dilution, anti-sheep, 1∶5000 dilution (SC-2004 and SC-2701 respectively, Santa Cruz Biotechnology, Santa Cruz, CA). Immunoreactive bands were visualized using enhanced chemiluminescence (ECL Plus, Amersham), band intensities were quantified using ImageJ imaging software (NIH), and they were normalized with GAPDH.

### Statistical Analysis

All data are presented as mean ± SEM. Statistical analysis was done by Student’s T-Test or one-way ANOVA with a Tukey’s post hoc test. P-values less than 0.05 were considered statistically significant. Statistical analyses were performed using Prism (GraphPad Software, Inc; version 5.0).

## Results

### Neurotransmitters and their Metabolites were Modulated in the Hippocampus and PFC

**Figure 1:**
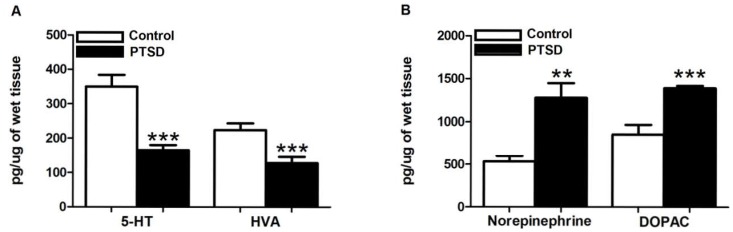
Hippocampus 5-HT, HVA, NE, and DOPAC levels post-stress. 5-HT and HVA were both significantly down-regulated (A), while NE and DOPAC were both significantly elevated (B), in the hippocampus in response to the predator exposure/psychosocial stress model. All data are presented as mean ± SEM. **p<0.001, ***p<0.0001 relative to the control group.

**Figure 2:**
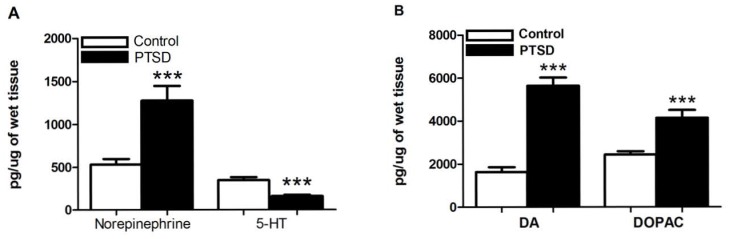
Prefrontal cortex 5-HT, NE, DA, and DOPAC levels post-stress. NE was elevated and 5-HT was down-regulated in the PFC in response to the predator exposure/psychosocial stress model (A). In addition, DA, and DOPAC were also significantly elevated (B). All data are presented as mean ± SEM. *p<0.05, **p<0.001, ***p<0.0001 relative to the control group.

To investigate the influence of the predator exposure/psychosocial stress regimen on neurotransmitter modulation, we examined endogenous levels of biogenic amines and their metabolites in the hippocampus and PFC of control and PTSD animals using HPLC (table 1). In the hippocampus, the levels of the tryptamine 5-HT and the DA metabolite HVA (figure 1A) were significantly lower in the PTSD group vs. controls, t(18) = 4.96, p<0.0001 and t(18) = 3.61, p<0.05, respectively. Conversely, the levels of the catecholamine NE and the DA metabolite DOPAC (figure 1B) were significantly higher in the PTSD group, t(18) = 4.05, p<0.001, and t(18) = 4.56, p<0.001, respectively. There were no significant changes noted in DA or 5-HIAA. In the PFC, the tryptamine 5-HT was significantly lower, while the catecholamine NE was higher (figure 2A), in the PTSD group vs. controls, t(18) = 2.25, p<0.05, and t(18) = 3.89, p<0.001. In addition, the levels of the catecholamine DA and the DA metabolite DOPAC (figure 2B) were significantly higher in the PTSD group, t(18) = 8.99, p<0.0001, and t(18) = 4.21, p<0.001 respectively. There were no significant changes noted in HVA or 5-HIAA.

**Table 1:** Changes in the levels of biogenic amines and metabolites in the PFC and hippocampus after the 31-day predator exposure/psychosocial stress regimen.

Parameters	Prefrontal Cortex	Hippocampus
5-HT		
Control	3250.2±503.3	349.8±34.0
PTSD	2067.9±148.2*	165.3±14.9***
NE		
Control	421.5±32.8	533.0±64.6
PTSD	671.6±55.3**	1277±172.3***
DA		
Control	1637.4±226.7	545.3±95.6
PTSD	5640.1±383.4***	646.4±74.5
HVA		
Control	1419.6±202.5	224.6±19.3
PTSD	1386.2±119.9	128.7±18.3**
DOPAC		
Control	2446.8±152.0	853.9±114.4
PTSD	4135.7±371.6***	1391.0±27.9***
5-HIAA		
Control	1907.6±253.4	976.1±152.7
PTSD	1525.2±175.4	854.1±63.6

Average concentration in pg/µg of wet tissue (±SEM) in the hippocampus (n = 10 for both groups).

5-HT: 5-hydroxytrypamine, HVA: homovanillic acid, NE: norepinephrine, DOPAC: 3,4-Dihydroxyphenylacetic acid, DA: dopamine, 5-HIAA: 5-Hydroxyindoleacetic acid.

*p<0.05,

**p<0.001,

***p<0.0001 relative to the control group.

### Rate-limiting Enzyme Fluctuations Confirmed Neurotransmitter Modulation

**Figure 3:**
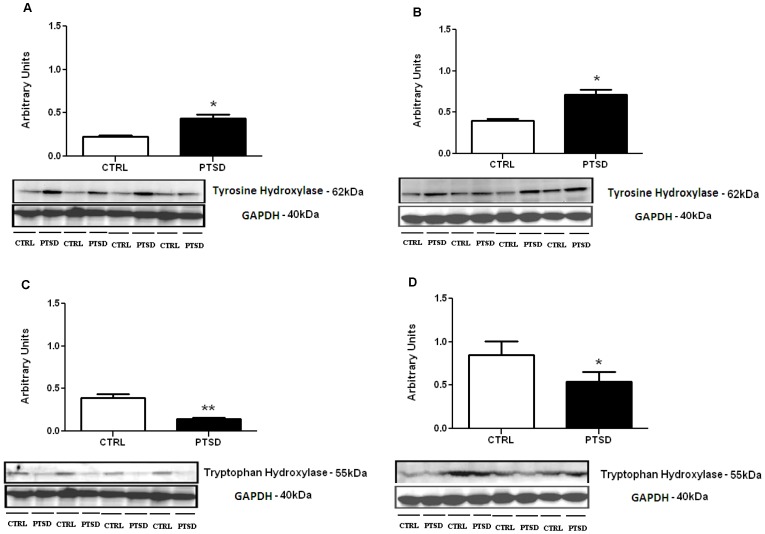
Rate-limiting enzymes tyrosine hydroxylase (catecholamines) and tryptophan hydroxylase (5-HT) post-stress. Tyrosine hydroxylase elevation in the PFC (A) and hippocampus (B) substantiated the findings of elevated levels of NE and DA, while down-regulated tryptophan hydroxylase in the PFC (C) and hippocampus (D) confirmed decreased levels of 5-HT. All data are presented as mean ± SEM. *p<0.05, **p<0.001.

To verify the observed changes in neurotransmitter levels in the hippocampus and PFC, we performed Western Blots of the rate-limiting enzymes for dopamine and norepinephrine (tyrosine hydroxylase (TH)) and 5-HT (tryptophan hydroxylase (TPH)). In both the hippocampus t(6) = 5.00, p<0.01 and PFC t(6) = 4.75, p<0.01, TH was elevated in the PTSD group vs. controls (figures 3A and 3C). Conversely, TPH was significantly downregulated in the hippocampus t(6) = 2.14, p<0.05 and PFC t(6) = 5.20, p<0.01 in the PTSD group vs. controls (figures 3B and 3D).

## Discussion

The present study sought to analyze neurotransmitter modulation in the hippocampus and PFC of rats subjected to pre-clinical PTSD via a predator exposure/psychosocial stress regimen. A myriad of animal models of PTSD exist, but the model by Zoladz et al. has been shown to cause heightened anxiety, exaggerated startle response, impaired cognition, and increased cardiovascular reactivity [25], [26], all of which are common symptoms reported in humans with PTSD [30], [31]. Although animal models have their limitations, a major component missing from human PTSD research is the ability to ascertain physiological data directly from specific brain regions immediately after a stressful event. The majority of the human physiological data gathered *in vivo* are derived from CSF, blood, or urine, which may not accurately reflect neurotransmitter modulation in the brain and certainly cannot distinguish between changes in specific brain regions. We have successfully obtained such data with this model, and to our knowledge we are the first to report the modulation of biogenic amines in the brains of PTSD animals. Two novel and important findings emerged from this study. First, the predator exposure/psychosocial stress regimen of pre-clinical PTSD produced measureable changes in neurotransmitters in the rat brain. Second, and possibly most important, 5-HT decreased and NE increased in both the hippocampus and PFC, providing evidence that the neurotransmitters previously implicated in PTSD pathophysiology are in fact modulated in response to persistent stressors.

Human PTSD research has made significant advances, but certain undefined variables still exist. Inconsistencies in patient backgrounds, types of stressful events (e.g., combat, rape, natural disasters, etc.), innumerable epigenetic differences, and the inability to obtain physiological data before PTSD development all present challenges. An animal model mitigates these variables, but as there are multiple animal models used in PTSD research with varied approaches and methods, careful selection is necessary. For our experiments, it was important to select an animal model of PTSD that matched, as closely as possible, the behavioral, psychological, and physiological elements of PTSD in humans. The predator exposure/psychosocial stress model by Zoladz et al. possesses both predictive and construct validity, making the model sensitive to clinically effective pharmacologic agents and the rationale underlying the model displays similarities to human PTSD [32]. This model demonstrates three hallmark features of PTSD: hormonal abnormalities, a long-lasting traumatic memory, and persistent anxiety [25], [26].

The modulation of various neurotransmitters observed with the predator exposure/psychosocial stress model is in concert with many of the neurotransmitter changes seen in human PTSD patients [14], [18], [21], [24]. Previous research has shown that stress blocks hippocampal long-term potentiation (LTP) and impairs its function [33]. The hippocampus, the primary region for spatial and long-term memory storage, expresses all of the 5-HT receptor families and reflects the overall serotonergic functions relating to cognition and mood in this region [34]. During stress, glucocorticoid production can reduce the excitability of hippocampal neurons, and 5-HT may have a protective effect against such damage by activating 5-HT_1A_ receptors [35]. Persistent activation of the HPA axis and excessive production of glucocorticoids, however, may directly reduce hippocampal 5-HT levels and adversely affect normal serotonergic transmission, thus contributing to heightened fear, depressed mood, and reduced resilience. The hippocampus also contains multiple NE receptors, which, when activated during stress, may contribute to the reinforcement of long-term memories [36]. In a study by Geracioti et al. involving male combat veterans with PTSD, CSF concentrations of NE were significantly higher vs. controls [18]. This finding could possibly explain why memories formed during extremely stressful events persist over time. Other evidence of catecholamine dysregulation in PTSD includes elevated urine catecholamine excretion, exaggerated biochemical responses to yohimbe, and clinical efficacy of adrenergic blockers [19]. Noradrenergic modulation was also noted with previous experiments utilizing the predator exposure/psychosocial stress animal model and treatments with selective serotonin reuptake enhancers (SSRE), α_2_ agonists, and tricyclic antidepressants [37]. Although we found no significant difference in DA levels in the hippocampus, reduction of HVA level was consistent with current human PTSD research [24]. HVA is a downstream product of DA metabolism, and traumatic stress may impede CNS release of DA from the substantia nigra and ventral tegmental area (VTA) [38], the primary CNS regions of dopaminergic neurons, thus reducing metabolite concentration. Another explanation may be that the majority of DA is directly converted to NE and not HVA, which also would further explain the marked increase in NE.

The PFC, responsible for executive functions such as consequences, drive, and social “control”, is highly innervated by serotonergic neurons from the raphe nuclei and also expresses an abundance of 5-HT receptors. The serotonergic neurons and 5-HT receptors, specifically the 5-HT_1A_ and 5-HT_2A_ receptors, are a key modulator of the PFC-amygdala corticolimbic circuit involved in threat and emotional responses [39]. PTSD-related aberrancies in this serotonergic system may cause inappropriate or incomplete extinction of conditioned fear. The PFC also contains NE receptors and receives input from NE neurons from the locus coeruleus, which are activated during the stress response [40]. Pathogical or stress-related elevations of NE in the PFC, however, may inhibit working memory and performance [41]. Current neuroimaging research indicates the PFC is hyporesponsive during symptomatic PTSD states and responsiveness is inversely proportional to symptom severity [42]. Whether marked elevations in NE directly or indirectly diminish PFC responsiveness and subsequent performance on cognitive emotional tasks remains unclear. In contrast to unchanged DA levels in the hippocampus, DA levels were significantly increased in the PFC, which was similar to the CSF and urine DA elevations seen in humans with PTSD[21]–[23]. In a similar manner to NE, stress-related elevations of DA may also impair working memory and performance. The PFC is densely innervated by dopaminergic neurons from the VTA, and dopamine release can be achieved via VTA or local stimulation. A recent study by Butts et al. demonstrated that stress-induced glucocorticoid stimulation of DA neurons caused a local release of DA in the PFC [43]. These data support the theory that overstimulation of the HPA axis and the resulting elevation in glucocorticoid activity can directly modulate DA and possibly other neurotransmitters.

## Conclusion

We utilized a predator exposure/psychosocial stress animal model of PTSD to determine if neurotransmitters were modulated in the rat hippocampus and PFC. We found that various neurotransmitters implicated in PTSD pathophysiology were altered in similar manners to those previously described in human PTSD research. In addition, we determined that other neurotransmitters were differentially expressed in the hippocampus and PFC. It would be an oversimplification, nonetheless, to presume that neurotransmitter modulation is the sole causal or resultant factor in PTSD development, as HPA axis, sympathoadrenal medullary pathway, and immune system alterations may also play an integral role in the pathophysiology of this complex disorder. Our use of the model established by Zoladz et al., [25], [26] produced results consistent with current neurotransmitter levels obtained via CSF and urine analysis in humans, further validating the efficacy of the model. Overall, our results demonstrate that there are CNS-specific modifications in neurotransmitter activity and expression in response to predator exposure/psychosocial stress. Future studies by our lab will investigate neurotransmitter modulation in the hippocampus and PFC in response to FDA-approved pharmacologic interventions and possibly provide insight into to why selective serotonin reuptake inhibitors are only moderately effective in many PTSD patients.
